# Apalutamide treatment and refractory hypothyroidism: effects of apalutamide on levothyroxine metabolism

**DOI:** 10.1530/ETJ-25-0185

**Published:** 2025-09-12

**Authors:** Florie Quattrochi, Solange Grunenwald, Philippe Caron

**Affiliations:** Department of Endocrinology and Metabolic Diseases, Cardiovascular and Metabolic Unit, Larrey University Hospital, Toulouse, France

**Keywords:** apalutamide, hypothyroidism, absorption, levothyroxine, UDP-glucuronosyltransferase

## Abstract

Apalutamide, a selective androgen receptor antagonist, is a new treatment for prostate cancer. Among the side effects observed during apalutamide treatment, an increased risk of hypothyroidism has been reported, particularly in patients previously treated with levothyroxine compared with untreated patients. Apalutamide is thought to stimulate hepatic UDP-glucuronosyltransferase activity, resulting in increased clearance and stimulation of the thyroxine enterohepatic cycle. A 65-year-old man on 150 μg/day levothyroxine treatment after total thyroidectomy for Graves’ disease was euthyroid. Treatment with apalutamide (240 mg/day) was started for metastatic prostate neoplasia. After 1 month, the TSH level was 47.9 μIU/mL, and the dose of levothyroxine was gradually increased. In the presence of refractory hypothyroidism (TSH 38 μIU/mL) despite 275 μg/day of levothyroxine (3.25 μg/kg/d), a levothyroxine absorption test was performed: the basal concentration of total T4 was 5.8 μg/dL; after oral absorption of 1,000 μg of levothyroxine, total T4 concentration increased, peaking at 8.1 μg/dL after 2 h. The percentage absorption of levothyroxine was 27.1% (normal: >60%). After 14 h, total T4 concentration fell to 5.7 μg/dL before rising again to 8.5 μg/dL at 20 h. In the absence of further levothyroxine intake, the second peak of total T4 concentration may be related to stimulation of UDP-glucuronosyltransferase activity, with increased T4 solubility in the bile, released into the small intestine, and finally absorbed, with increased T4 concentration at 20 h in the patient, attesting to the stimulated T4 enterohepatic cycle during apalutamide treatment. Overall, the result of this clinical study suggests that apalutamide reduces the digestive absorption of levothyroxine, in addition to stimulating the activity of hepatic UDP-glucuronosyltransferase, explaining the higher prevalence of hypothyroidism during apalutamide treatment in patients previously treated with levothyroxine.

## Established facts

- Apalutamide can stimulate hepatic UDP-glucuronosyltransferase activity, resulting in increased clearance and stimulation of the thyroxine enterohepatic cycle.
- Of the side effects observed during apalutamide treatment, an increased risk of hypothyroidism has been reported, particularly in patients previously treated with levothyroxine.

## Novel insights

- Apalutamide reduces the digestive absorption of levothyroxine, explaining the higher prevalence of hypothyroidism in patients treated with levothyroxine.

## Introduction

Treatment with apalutamide, a new-generation anti-androgen, is indicated in the management of non-metastatic prostate cancer that is resistant to chemical castration and metastatic prostate cancer. Although this treatment is generally well tolerated, it has some side effects, particularly hypothyroidism. The SPARTAN and TITAN studies described the development or worsening of hypothyroidism during apalutamide treatment, particularly in patients treated with levothyroxine (1, 2). However, observations in patients with pre-existing hypothyroidism treated with levothyroxine are poorly documented and raise important clinical questions about the monitoring and adjustment of thyroid replacement therapy in these patients.

This clinical study reports an euthyroid patient on levothyroxine treatment who developed refractory hypothyroidism with reduced levothyroxine absorption following treatment with apalutamide, suggesting that apalutamide decreases the digestive absorption of levothyroxine, in addition to stimulating the activity of hepatic UDP-glucuronosyltransferase (3), explaining the higher prevalence of hypothyroidism in patients previously treated with levothyroxine.

## Patient

The patient, aged 65 years, underwent total thyroidectomy in January 2024 for recurrent Graves’ disease with a large goiter, diagnosed in 2021 and treated with synthetic antithyroid drugs. Levothyroxine substitutive therapy was started immediately after surgery, resulting in euthyroidism with TSH concentrations below 4 μIU/mL until June 2024 (Table 1). The patient weighed 81 kg (BMI: 25.6) and was treated with 150 μg/day of levothyroxine (1.85 μg/kg/day).

**Table 1:** TSH and free T4 concentrations as a function of levothyroxine dose in a patient with refractory hypothyroidism before and during apalutamide treatment.

	Before treatment		During treatment						
	M-5	M-1	M+1	M+3	M+4	M+5	M+6	M+8	M+11
Levothyroxine dose (μg/day)	150	150	150	175	225	275	300	400	450
TSH (μIU/mL)	4.14	3.93	47.9	70	52.4	37.1	54	11	1.08
Free T4 (pmol/L)				7.7	10.3		8.1		

In the case of lymph node recurrence of prostate adenocarcinoma diagnosed in 2020, treatment with apalutamide (240 mg/day) combined with quarterly injections of triptorelin was started in July 2024.

In August 2024, TSH concentration was 47.9 μIU/mL with no clinical signs of hypothyroidism, and levothyroxine was increased to 175 μg/day. Despite the increase in levothyroxine dose, the TSH was 75.2 μIU/mL in October 2024, leading to an increase in levothyroxine to 225 μg per day. Levothyroxine was increased to 275 μg/day in November 2024 after the TSH remained elevated at 52.4 μIU/mL, still without clinical symptoms. The lipid profile in December 2024 showed total cholesterol of 2.44 g/L, with LDLc of 1.40 g/L and HDL of 0.89 g/L.

Despite increasing the levothyroxine dose, high TSH level to 52.4 μIU/mL, free T4 to 10.3 pmol/L (normal: 11.5–22.7) and free T3 to 2.9 pmol/L (normal: 3.5–6.5), assessment of levothyroxine malabsorption was performed, but no abnormalities were found: the conditions for treatment with levothyroxine were met, with levothyroxine tablets taken at a distance from apalutamide in the morning on an empty stomach. The patient was not taking calcium or iron supplements or a proton pump inhibitor. A urea breath test (Helikit) excluded *Helicobacter pylori* infection, and IgA transglutaminase, endomysium, and gliadin antibodies excluded gluten intolerance.

A levothyroxine absorption test was carried out to identify a disorder in the absorption of levothyroxine. After a fasting period of 8 h, thyroid hormone concentrations (total T4, free T4, free T3) were measured every 2 h for 24 h following an oral dose of 1,000 μg levothyroxine (4, 5, 6).

Given the basal TSH and free T4 concentrations of the levothyroxine absorption test, the dose of levothyroxine was increased to 300 μg/day, i.e., 3.6 μg/kg/day. In January 2025, the thyroid function evaluation showed a TSH level of 54 μIU/mL, a low free T4 concentration at 8.1 pmol/L (normal: 11.5–22.7), and a free T3 level of 3.8 pmol/L (normal: 3.5–6.5). The patient did not have any clinical signs of hypothyroidism. The dose of levothyroxine was increased to 400 μg/day (i.e. 4.8 μg/kg/d), and thyroid function test in March 2025 showed a TSH level of 11 μIU/mL. Then, the levothyroxine dose was 450 μg/day (i.e. 5.4 μg/kg/d) and TSH level was normal (1.08 μIU/mL) in June 2025 (Table 1).

## Methods

Plasma concentrations of TSH, total T4, free T4, and free T3 were determined by electrochemiluminescence immunoassay (ECLIA, Laboratoire Roche Diagnostics, France).

For the levothyroxine absorption test (4, 5, 6), 1,000 μg of levothyroxine was given orally after an 8 h fast. TSH level was measured at T0 and T24 h, while thyroid hormone concentrations (total T4, free T4, free T3) were measured every 2 h for 24 h.

The percentage absorption of levothyroxine was calculated using the formula: total T4 increment (peak total T4 – basal total T4) (μg/dL) × 10 × 0.442 × BMI × 100/dose of levothyroxine (μg). Normal absorption of levothyroxine is greater than 60%.

## Results

After a total thyroidectomy, the patient was treated with levothyroxine 150 μg/day, with a TSH level of 4.14 μIU/mL in March 2024 and 3.95 μIU/mL in June 2024, indicating euthyroidism. After initiation of apalutamide treatment in July 2024, an increase in TSH was observed, with a maximum of 70 μIU/mL in October 2024 (3 months after initiation of apalutamide treatment), despite the significant increase in levothyroxine dose (Table 1).

**Figure 1:**
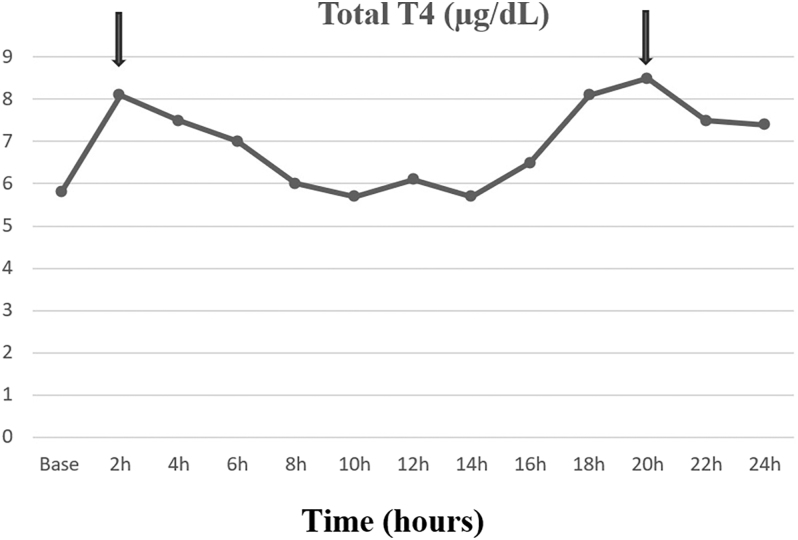
Changes in total T4 concentration during the levothyroxine absorption test of the patient treated with apalutamide.

**Figure 2:**
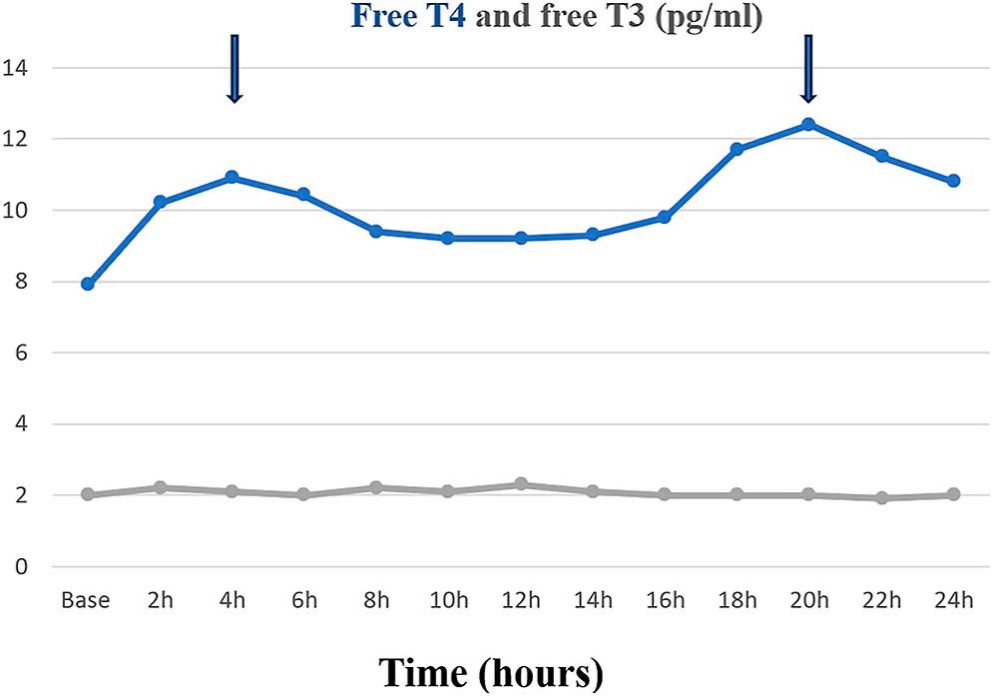
Changes in free T4 and free T3 concentrations during the levothyroxine absorption test of the patient treated with apalutamide.

During the levothyroxine absorption test, baseline TSH concentration was 38 μIU/mL and 32 μIU/mL 24 h after oral absorption of 1,000 μg of levothyroxine. The basal concentration of total T4 was 5.8 μg/dL, with an initial peak of 8.1 μg/dL 2 h after the levothyroxine absorption; the percentage absorption of levothyroxine was 27.1% (normal: >60%). Total T4 then fell to a low concentration of 5.7 μg/dL at 14 h before rising again to a second peak of 8.5 μg/dL at 20 h (Fig. 1). The profile of free T4 was similar to that of total T4, with a baseline of 7.9 pg/mL and an initial peak of 10.9 pg/mL at 4 h. Free T4 level then fell to 9.2 pg/mL at 10 h. It then rose again, with a second peak of 12.4 pg/mL at 20 h (Fig. 2). The concentration of free T3 was low and did not vary significantly after levothyroxine absorption (Fig. 2).

## Discussion

Apalutamide is a selective androgen receptor inhibitor indicated for the treatment of hormone-sensitive and metastatic prostate cancer associated with castration or treatment to inhibit androgen secretion, or resistant to chemical castration with a high risk of metastatic progression (7). It is usually prescribed at an average dose of 240 mg/day.

Among the side effects observed during treatment with apalutamide, hypothyroidism was reported in 8.1% of patients in the apalutamide group compared to 2% in the placebo group in the SPARTAN study (1). In the TITAN study, hypothyroidism was reported in 6.5% of patients in the apalutamide arm versus 1.1% in the placebo arm (2). Apalutamide, a potent CYP3A4 inducer, is thought to stimulate the hepatic activity of UDP-glucuronyl transferase via activation of the PXR nuclear receptor, leading to increased clearance of T4 with a decrease in its systemic concentration and hypothyroidism (3). After levothyroxine absorption at time 0, hormonal monitoring of the patient treated with apalutamide shows that total and free T4 concentrations display a second peak at 20 h, which, in the absence of further levothyroxine intake, can be related to activation of hepatic UDP-glucuronyl transferase and stimulation of the thyroxine enterohepatic cycle.

However, this effect of apalutamide on hepatic UDP-glucuronyl transferase does not explain the higher incidence of hypothyroidism in patients already treated with levothyroxine. In the SPARTAN trial, hypothyroidism was present in 30% of patients treated with levothyroxine replacement therapy in the apalutamide group compared with 3% in the placebo group (1). During the patient’s levothyroxine absorption test, total T4 and free T4 concentrations show an early peak at 2 and 4 h, respectively, which is related to the digestive absorption of levothyroxine. However, the percentage absorption of levothyroxine was 27.1% (normal >60%), indicating a reduction in the digestive absorption of levothyroxine in patients treated with apalutamide. Apalutamide might impair levothyroxine absorption by a direct effect or mediated via changes in gut motility, microbiota, or transporter expression at the level of the small intestine. Further studies are needed to clarify the pathophysiological mechanism of the decrease in the digestive absorption of levothyroxine during apalutamide treatment.

During treatment with apalutamide, the doses of levothyroxine required to restore normal thyroid function are higher in patients with pre-existing hypothyroidism than in patients without a history of thyroid disease. In the series by Moffatt *et al.* (8), the mean dose of levothyroxine in patients with pre-existing hypothyroidism increased from 1.14 μg/kg/day before apalutamide treatment to 1.82 μg/kg/day during apalutamide treatment. In patients with no history of thyroid disease, the mean dose of levothyroxine during apalutamide treatment was 0.94 μg/kg/day (8).

In addition to poor compliance, a classic and common cause of refractory hypothyroidism in patients treated with oral levothyroxine, reduced digestive absorption of levothyroxine may be secondary to reduced gastric acidity (autoimmune gastritis, treatment with proton pump inhibitors), a high-fiber diet, or iron or calcium salts supplementation with reduced bioavailability of levothyroxine. Less commonly, autoimmune or vascular damage of the intestinal mucosa may be responsible for reduced absorption of levothyroxine (9).

It should be noted that the mean time between discontinuation of apalutamide therapy and the first decrease in TSH concentration is 11.7 weeks in patients with pre-existing hypothyroidism (with a higher dose of levothyroxine than before treatment) and 10.4 weeks in patients without pre-existing thyroid pathology (8).

Further studies are needed to assess the long-term recovery of thyroid function in patients after discontinuation of apalutamide treatment.

## Conclusion

In the patient treated with levothyroxine for postoperative hypothyroidism, the levothyroxine absorption test shows a decrease in the digestive absorption of levothyroxine and activation of hepatic UDP-glucuronyl transferase with stimulation of the thyroxine enterohepatic cycle during treatment with apalutamide.

Based on a case series of patients, recommendations for thyroid management before, during, as well as after the interruption of apalutamide treatment have been recently published in this Journal (10).

Clinically, TSH monitoring before starting apalutamide treatment is recommended in the management of these patients. It is important to monitor thyroid function (TSH) in patients treated with apalutamide, especially if they are being treated with oral levothyroxine for pre-existing thyroid insufficiency. Some authors recommend monitoring TSH every 2–4 months during apalutamide treatment (1, 2, 11), although it should be noted that the increase in TSH may occur early after initiation of apalutamide treatment, as early as the first month in the reported patient.

Finally, after withdrawal of apalutamide treatment, TSH levels should be monitored in order to adjust the levothyroxine dosage (before returning to pre-therapeutic doses) and to prevent the onset of iatrogenic thyrotoxicosis.

## Declaration of interest

The authors declare that there is no conflict of interest that could be perceived as prejudicing the impartiality of the work reported.

## Funding

This research did not receive any specific grant from any funding agency in the public, commercial, or not-for-profit sector.

## Author contribution statement

FQ collected the clinical data and wrote the manuscript. SG took care of the patient, analyzed the data, and reviewed the manuscript. PC conceptualized of the study, reviewed the manuscript, and edited the article.

## Patient consent

Written informed consent has been obtained from the patient for publication of this case report.
